# Sodium Humate Combined with Low-Dose Cefixime Alleviates Intestinal Injury in ETEC Infection via Inhibition of the TLR4/NF-κB Pathway

**DOI:** 10.3390/biom16060814

**Published:** 2026-05-30

**Authors:** Xingyao Liu, Danning Tong, Yun Liu, Shengzi Jin

**Affiliations:** Heilongjiang Provincial Key Laboratory of Pathogenic Mechanism for Animal Disease and Comparative Medicine, College of Veterinary Medicine, Northeast Agricultural University, Harbin 150030, China; liuxingyao@neau.edu.cn (X.L.); tongdanning@neau.edu.cn (D.T.)

**Keywords:** sodium humate, cefixime, enterotoxigenic *Escherichia coli*, intestinal barrier, TLR4-NF-κB pathway

## Abstract

This study aimed to evaluate the protective effects of sodium humate (HNa) alone and in combination with low-dose cefixime (CFM) in mice infected with enterotoxigenic *Escherichia coli* (ETEC). An ETEC infection mouse model was established to compare the effects of individual or combined interventions on physiological parameters, intestinal morphology, barrier function, levels of specific intestinal bacterial groups, cell proliferation/apoptosis, and inflammatory pathways. The results showed that the HNa + CFM combination significantly promoted body weight recovery, ameliorated damage to jejunal villus structure and ultrastructure, and increased the mRNA expression of mucins (*MUC1/2/3*) and tight junction proteins (*ZO-1*, *Occludin*, *Claudin-1*) compared to the ETEC group. Concurrently, the combined treatment significantly reduced fecal *E. coli* counts and increased the abundance of *Lactobacillus* and *Bifidobacterium*, promoted epithelial repair by upregulating proliferation-related genes (*EGFR*, *PCNA*, *TGF-β1*), and decreased the *Bax*/*Bcl-2* ratio. Furthermore, the combined intervention significantly reduced serum LPS levels and consequently suppressed ETEC-induced activation of the TLR4/MyD88/NF-κB pathway, as evidenced by reduced protein expression of TLR4 and MyD88, decreased phosphorylation of IκBα and p65, and diminished nuclear accumulation of NF-κB p65, leading to downregulation of pro-inflammatory cytokines (TNF-α, IL-6, IL-1β) and elevation of *IL-10*. In conclusion, the combined application of HNa and low-dose CFM showed additional protective benefits against ETEC infection. These effects were associated with multi-targeted repair of the intestinal barrier, modulation of measured bacterial levels, and suppression of excessive inflammatory responses. This strategy offers a potential approach for the clinical management of bacterial enteritis and reducing antibiotic dependence.

## 1. Introduction

Enterotoxigenic *Escherichia coli* (ETEC) is one of the pathogens responsible for neonatal diarrhea and bacterial diarrhea in young animals [1]. Its K88 serotype has drawn significant attention due to its high prevalence and wide distribution [2]. The pathogenicity of ETEC primarily involves colonization factors and adhesins at the tip of flagella, which enable attachment to intestinal epithelial cells in the small intestine. During growth and proliferation, ETEC produces heat-labile toxin and heat-stable toxin [3]. These enterotoxins disrupt the secretory function of enterocytes, leading to excessive secretion and accumulation of water and electrolytes in the intestinal lumen, ultimately resulting in diarrhea [4]. Studies have shown that ETEC colonization and toxin action directly damage intestinal villus structure and tight junctions, compromising the physical barrier of the intestinal mucosa and increasing intestinal permeability. This not only facilitates the translocation of endotoxins, such as LPS but also triggers significant local and even systemic inflammatory responses, exacerbating tissue damage [5]. In animal husbandry, ETEC infections cause substantial economic losses in piglet and calf production, as manifested by growth retardation, reduced feed conversion efficiency, and increased mortality [6]. For a long time, antibiotics have been the primary means of controlling this disease. However, the increasing multidrug resistance of ETEC strains has become a major challenge for global public health and animal health [5]. Therefore, exploring novel, effective, and resistance-resistant alternative or adjunctive therapeutic strategies has become an urgent research priority.

Faced with this challenge, the combined application of drugs with different mechanisms of action is regarded as a potential solution. Cefixime (CFM) is an orally effective third-generation cephalosporin antibiotic that exhibits excellent antibacterial activity against *E. coli*, *Streptococcus pyogenes*, *Streptococcus pneumoniae*, and other bacteria [7,8]. However, the sole use of antibiotics may exacerbate intestinal flora dysbiosis while clearing pathogens and offers limited repair for existing damage to the intestinal barrier structure. Sodium humate (HNa) is a sodium salt of humic substances extracted from natural resources such as weathered coal, lignite, or peat. Its core structure consists of aromatic macromolecules rich in active functional groups such as carboxyl and phenolic hydroxyl groups [9,10]. Due to its strong chelating, redox, and adsorption capacities, as well as its richness in various bioactive components, HNa demonstrates multiple pharmacological effects, including antibacterial, anti-inflammatory, antioxidant, and anti-diarrheal properties [11,12]. Early studies indicated that dietary supplementation with HNa improved growth performance, immune function, and antioxidant capacity, and reduced the incidence of diarrhea in weaned piglets [10]. Our preliminary research has shown that HNa can significantly ameliorate intestinal barrier function in enteritis induced by *E. coli* infection [13]. The study demonstrated that the combination of propolis and low-dose CFM enhanced antibacterial efficacy, allowing effective treatment at lower antibiotic doses [14]. Additionally, studies have reported that the combined use of humic acid and lincomycin in the diet significantly improved the condition of broilers infected with *Clostridium* [15]. However, there remains a lack of green and effective therapeutic approaches for intestinal diseases caused by ETEC infection. This study explored the combined use of low-dose CFM and HNa in a mouse model of ETEC infection to preliminarily assess its feasibility and protective effects, as well as to investigate potential mechanisms. The intervention simultaneously addresses antibacterial activity and intestinal barrier protection, thereby helping to control infection and promote intestinal recovery, may provide a potential experimental strategy for managing ETEC infection while reducing antibiotic dependence.

## 2. Materials and Methods

### 2.1. Main Reagents

HNa was purchased from the Institute of Chinese Academy of Sciences (Taiyuan, China). Its composition on a dry matter basis included 75.00% humic acids, 20.52% ash, and 4.48% water-soluble substances; it also contained 14.22% moisture (on an air-dried basis). CFM was obtained from Beijing Bio-Leap Biotechnology Co., Ltd. (Beijing, China). The ETEC K88 strain was preserved in our laboratory [13]. ETEC K88 was inoculated into LB liquid medium and cultured at 37 °C until the logarithmic growth phase. Serial dilutions of the bacterial suspension were prepared, and the optical density at 600 nm (OD600) was measured for each dilution. Meanwhile, an aliquot of each dilution was spread onto LB agar plates, and after incubation, the colony-forming units (CFU) were counted. A linear regression standard curve was established using OD600 as the independent variable and CFU/mL as the dependent variable. The bacterial suspension was centrifuged at 5000× *g* for 10 min, and the bacterial pellet was collected and washed three times with phosphate-buffered saline. Based on the standard curve, the bacterial concentration was calculated by measuring the OD600 value, and the final suspension was diluted with PBS to 5 × 10^10^ CFU/mL for subsequent use.

### 2.2. Mouse Model Establishment and Drug Treatment

4-week-old (weight: 26 ± 2 g) specific-pathogen-free Kunming mice were obtained from Liaoning Changsheng Biotechnology Co., Ltd (Benxi, China). The mice were maintained under standardized environmental conditions (22–24 °C, 50–60% relative humidity, 12 h/12 h light/dark cycle) with unrestricted access to food and water. The 3R principles were followed in the use of laboratory animals. The method used complied with the ARRIVE guidelines and the National Research Council’s recommendations regarding the Care and Use of Laboratory Animals. All protocols were approved by the Laboratory Animal Ethics Committee of Northeast Agricultural University on 6 March 2023 (approval number: NEAUEC20230338), ensuring compliance with ethical standards for animal research.

**Figure 1:**
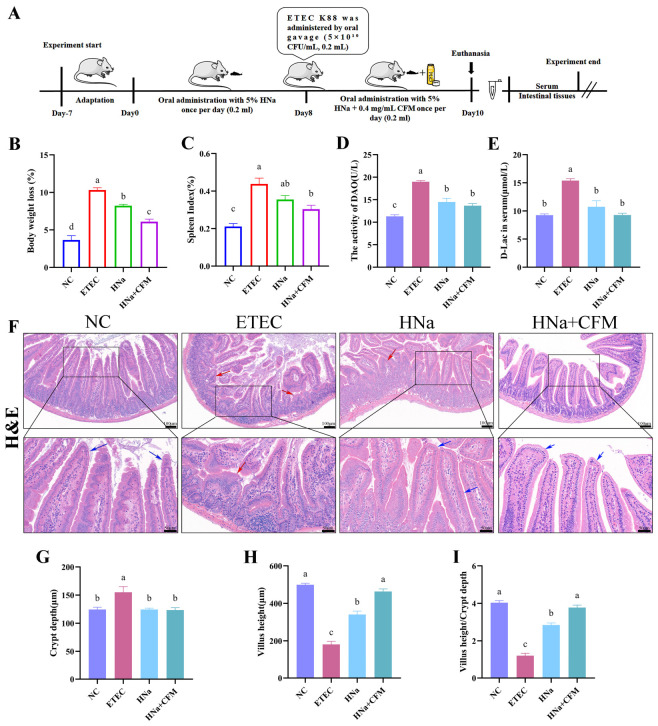
Effect of HNa combined with CFM on ETEC-infected mice. (**A**) The specific experimental procedure; (**B**) The body weight loss (body weight loss (%) was calculated relative to the body weight before ETEC challenge); (**C**) Mouse spleen index; (**D**,**E**) Serum DAO and D-lac levels in mice; (**F**) Representative H&E-stained images of the jejunum; scale bar = 100 μm/50 μm (red arrows indicate damaged intestinal villi, blue arrows indicate normal villi, *n* = 3); (**G**–**I**) Crypt depth, villus height, and the villus height to crypt depth ratio in the mouse jejunum. Data are presented as the mean ± SEM, *n* = 7. Different lowercase letters above the bars denote statistically significant differences (*p* < 0.05). The absence of a letter difference indicates no statistical significance (*p* > 0.05).

After a one-week acclimatization period with ad libitum access to food and water, the experiment commenced. The 28 mice were randomly divided into four groups (*n* = 7): negative control (NC, saline-treated), model (ETEC), HNa intervention (HNa), and HNa combined with CFM intervention (HNa + CFM). The experimental design is shown in Figure 1A. NC and ETEC groups: 0.2 mL of saline daily by oral gavage; HNa and HNa + CFM groups: 0.2 mL of 5% HNa solution daily. On day 8, the ETEC, HNa, and HNa + CFM groups were orally challenged with 0.2 mL ETEC K88 suspension (5 × 10^10^ CFU/mL), corresponding to 1 × 10^10^ CFU per mouse. This dose was selected based on previously established acute ETEC K88-induced intestinal injury models in mice and was intended to produce reproducible intestinal damage for evaluating the protective effects of the interventions [1,16]. Prior to ETEC infection, mice were fasted for 12 h and deprived of water for 4 h. To reduce gastric acid interference, cimetidine (50 mg/kg) was injected intraperitoneally 1 h before infection. At 12 h post-infection, mice in the HNa + CFM group began receiving 0.2 mL of 0.4 mg/mL CFM solution by oral gavage, which continued for 3 days. The concentration of HNa used was based on the results of preliminary studies from our research group [13]. Mice were anesthetized with isoflurane, blood samples were collected, then the mice were euthanized by cervical dislocation, and the abdominal cavity was opened aseptically for sampling.

### 2.3. Histopathological Examination

Following prefixation, jejunal tissues were dehydrated through a graded ethanol series, cleared with xylene, and embedded in paraffin. Sections of 3–5 μm thickness were prepared, stained with hematoxylin and eosin (H&E, Wuhan Servicebio Technology Co., Ltd., Wuhan, China), and examined under a light microscope. Images were captured and analyzed using a pathological image analysis system (BX-FM; Olympus Corp, Tokyo, Japan).

### 2.4. Observation of Intestinal Tissue Ultrastructure

Jejunal samples were trimmed, then fixed overnight in pre-chilled 2.5% glutaraldehyde. After rinsing, the tissues were post-fixed in 1% osmium tetroxide and rinsed again. Subsequently, they were dehydrated through a graded series of ethanol and acetone, infiltrated, and embedded in a mixture of acetone and epoxy resin. Finally, ultrathin sections were prepared, stained with uranyl acetate and lead citrate, and observed and imaged using a transmission electron microscope (Tecnai, Hitachi, Tokyo, Japan).

### 2.5. ELISA Analysis

Serum was collected after centrifugation of blood samples. Following the manufacturer’s instructions of the respective ELISA kits, the levels of DAO, D-lac, LPS (Beyotime Biotechnology, Shanghai, China), IL-1β, IL-6, and TNF-α (Boster Biological Technology, Wuhan, China) in the serum were measured. The results were then statistically analyzed.

### 2.6. Quantitative Real-Time PCR Analysis

Total RNA was isolated from jejunal tissues with a total RNA extraction kit (Takara Bio Inc., Shiga, Japan), strictly adhering to the manufacturer’s instructions. The isolated total RNA was subsequently converted into cDNA via a reverse transcription kit. The resulting cDNA was then used for qPCR to determine the relative mRNA expression levels of *Occludin*, *Claudin-1*, *ZO-1*, *E-cadherin*, *β-catenin*, *MUC1*, *MUC2*, *MUC3*, *TNF-α*, *IL-6*, *IL-1β*, *IL-10*, *PCNA*, *TGF-β1*, *EGFR*, *Bax*, *Bcl-2*, *TLR4*, and *NF-κB* in the jejunums (Roche 480 System, Roche, Basel, Switzerland). All qPCR primer sequences are provided in Table S1. Relative mRNA expression levels were determined via the 2^−ΔΔCt^ method, with β-actin used as the internal reference gene.

### 2.7. Western Blot Analysis

Jejunal tissue was used to isolate protein with RIPA lysis buffer. Protein samples were then subjected to denaturation by boiling in PAGE loading buffer. Following separation via SDS-PAGE and transfer onto membranes, the blots were blocked and then probed with primary antibodies targeting β-actin, Histone H3, Bax, Bcl-2, NF-κB, P-NF-κB, p-IκBα, IκBα, MyD88 and TLR4 (Wanlei Biotechnology, Shenyang, China), after which HRP-conjugated secondary a ntibodies were applied. Chemiluminescence was used to visualize the protein bands, with β-actin employed as the loading control.

### 2.8. Fecal Microbial Community Analysis

The composition of the microbial community in mouse rectal feces was analyzed using qPCR. The specific procedure followed the method described by Sabat et al. [17]. Total bacterial DNA was extracted from the rectal contents using the TIANamp Stool DNA Kit (Tiangen Biotech Co., Ltd., Beijing, China), and its concentration and purity were measured. The extracted total bacterial DNA and standard plasmids for *Escherichia coli*, *Bifidobacterium*, and *Lactobacillus* at different concentrations were subjected to qPCR amplification, respectively. Standard curves were constructed based on regression analysis between the concentrations of the standard plasmids and their corresponding Ct values. The Ct values of the samples were applied to the regression equations to calculate the DNA copy numbers of each target bacterial genus. Data are presented as the common logarithm of bacterial copy number per gram of content [lg(copies/g)]. Table S2 lists the sequences for the microbe-specific primers employed.

### 2.9. Statistical Analysis

All data are expressed as the mean ± SEM. Statistical comparisons were performed using one-way ANOVA followed by Tukey’s post hoc test for multiple comparisons. GraphPad Prism 8.0.1 (San Diego, CA, USA) was used to generate the statistical graphs. A value of *p* < 0.05 was considered statistically significant after adjustment for multiple comparisons.

### 2.10. AI-Assisted Language Improvement

During the preparation of this manuscript, the authors used DeepSeek (DeepSeek-V3, Hangzhou, China) solely for grammatical correction, spell-checking, punctuation revision, and language refinement. After using this tool, the authors carefully reviewed and edited the modified content as necessary and they assume full responsibility for the final content and integrity of this publication.

## 3. Results

### 3.1. Protective Effect of HNa Combined with CFM on ETEC-Infected Mice

As shown in Figure 1B, the body weight loss in the ETEC group was significantly higher than that in the NC group (*p* < 0.05), indicating that ETEC infection induced marked weight reduction in mice. Compared with the ETEC group, both the HNa and HNa + CFM intervention groups showed a significant decrease in body weight loss (*p* < 0.05). Moreover, the HNa + CFM group exhibited significantly lower body weight loss than the HNa group (*p* < 0.05), suggesting that the combined intervention more effectively alleviated ETEC-induced weight loss. These results indicate that HNa and CFM intervention can significantly promote the recovery of mouse body weight. Results for the spleen index (Figure 1C) showed a significant increase in the ETEC group compared to the NC group (*p* < 0.05). HNa intervention alone showed a non-significant trend toward reduction, whereas HNa + CFM significantly reduced the spleen index (*p* < 0.05). Changes in serum DAO and D-lac levels are key indicators of intestinal damage. Following ETEC infection, serum levels of DAO and D-lac were significantly elevated (*p* < 0.05; Figure 1D,E), indicating impaired intestinal barrier dysfunction. Intervention with HNa combined with CFM significantly reduced the levels of both DAO and D-lac (*p* < 0.05). We further examined histopathological sections of the jejunum (Figure 1F). After ETEC infection, the ETEC group exhibited varying degrees of jejunal villus shortening and disruption (indicated by red arrows). Statistical analysis revealed that, compared to the NC group, the ETEC group had significantly reduced jejunal villus height and villus height to crypt depth ratio (*p* < 0.05; Figure 1G–I). Compared to the ETEC group, both the HNa and HNa + CFM groups showed a significant increase in villus height and the villus height to crypt depth ratio (*p* < 0.05). Furthermore, the HNa + CFM group demonstrated a more pronounced restorative effect compared to the HNa group alone (*p* < 0.05). The above results demonstrate that HNa combined with CFM has a significant protective effect against intestinal damage induced by ETEC.

### 3.2. Protective Effects of HNa Combined with CFM on Ultrastructure and Mechanical Barrier in ETEC-Infected Mice

**Figure 2:**
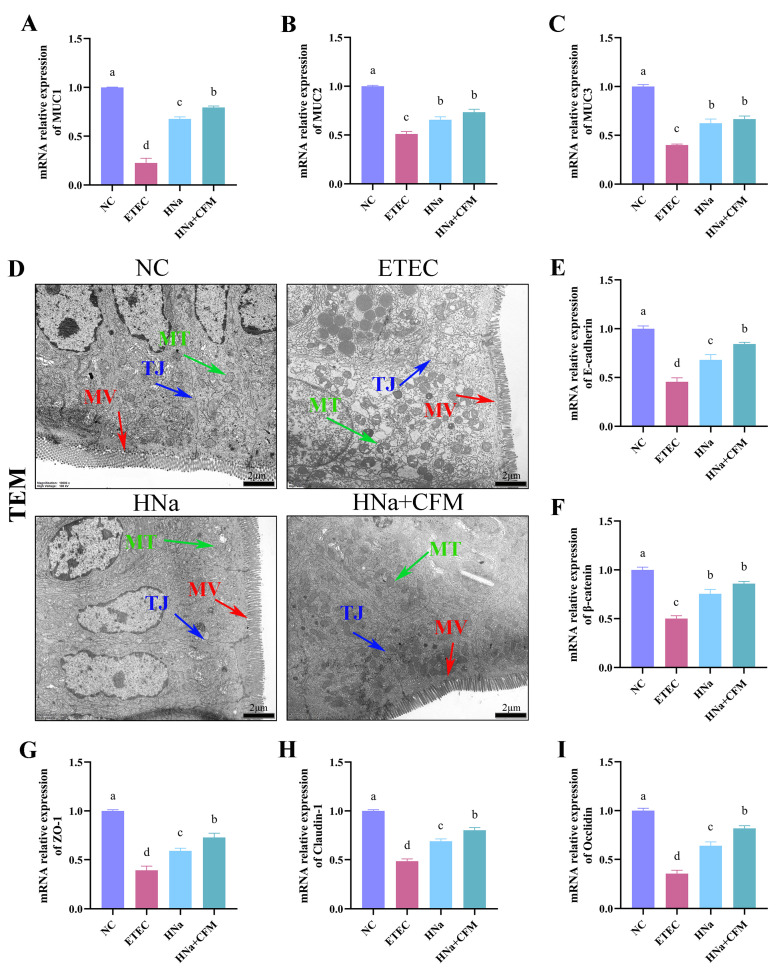
Effect of HNa combined with CFM on the ultrastructure and mechanical barrier of ETEC-infected mice. (**A**–**C**) Quantitative RT-PCR analysis of *MUC1*, *MUC2*, and *MUC3* mRNA levels in the jejunum. (**D**) Morphology of the jejunum observed by transmission electron microscopy (2 µm). (MV: microvilli; TJ: tight junction; MT: mitochondria, *n* = 3). (**E**–**I**) Quantitative RT-PCR analysis of *E-cadherin*, *β-catenin*, *ZO-1*, *Occludin*, and *Claudin-1* mRNA levels in the jejunum. Data are presented as the mean ± SEM, *n* = 6. Different lowercase letters above the bars denote statistically significant differences (*p* < 0.05). The absence of a letter difference indicates no statistical significance (*p* > 0.05).

As shown in Figure 2A–C, compared to the NC group, the mRNA expression of *MUC1*, *MUC2*, and *MUC3* in the jejunum was significantly decreased following ETEC infection (*p* < 0.05). Intervention with HNa combined with CFM significantly promoted the mRNA expression of *MUC1*, *MUC2*, and *MUC3* (*p* < 0.05), indicating that HNa combined with CFM can significantly restore ETEC-induced damage to the jejunal mucus layer. Simultaneously, observations under transmission electron microscopy (Figure 2D) revealed that ETEC infection led to disruption and reduction in intercellular tight junctions (indicated by blue arrows). Intestinal epithelial cells displayed abnormal morphology with a noticeable decrease in mitochondria, which exhibited loose, irregular, or fragmented cristae (indicated by green arrows). Microvilli were markedly sparse, showing varying degrees of breakage and shedding, indicating severe intestinal structural damage (indicated by red arrows). After HNa intervention, intercellular tight junctions became clear, intestinal epithelial cell edema was alleviated, mitochondria increased in number, and microvilli were of moderate length and orderly arrangement. In the HNa combined with CFM intervention group, intercellular tight junctions were intact and clear, intestinal epithelial cells were aligned and regular in shape, mitochondria were normal in morphology and significantly increased in number, and microvilli were neatly arranged. Furthermore, assessment of mRNA levels for adhesion and tight junction proteins showed that, compared to the NC group, the mRNA expression levels of *E-cadherin* and *β-Catenin* (Figure 2E,F) were significantly lower in the ETEC group (*p* < 0.05). Their expression levels were significantly increased after intervention with HNa combined with CFM (*p* < 0.05). Compared to the NC group, the mRNA expression levels of *ZO-1*, *Occludin*, and *Claudin-1* were all significantly reduced in the ETEC group (*p* < 0.05) (Figure 2G–I). Both HNa alone and HNa combined with CFM interventions significantly increased the mRNA expression of *ZO-1*, *Occludin*, and *Claudin-1* (*p* < 0.05). Additionally, compared to HNa intervention alone, the HNa + CFM group showed a more significant increase in the mRNA expression of *ZO-1*, *Occludin*, and *Claudin-1* (*p* < 0.05). This further indicates that HNa combined with CFM has a protective effect against ETEC-induced damage to adherens junctions and tight junctions in mice. The above results demonstrate that administration of HNa combined with CFM provides significant protection against ETEC-induced damage to intestinal ultrastructure and the mechanical barrier.

### 3.3. Effects of HNa Combined with CFM on Specific Gut Bacterial Levels in Feces of ETEC-Infected Mice

**Figure 3:**
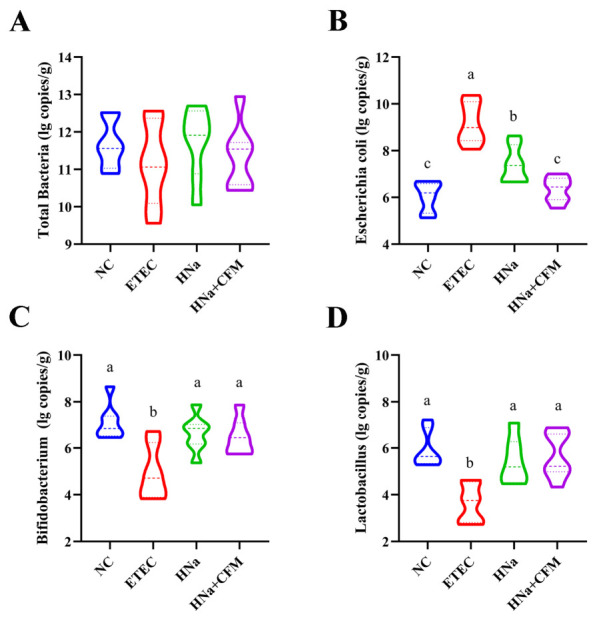
Effects of HNa combined with CFM on gut bacterial levels in ETEC-infected mice. (**A**–**D**) Levels of total bacteria, *E. coli*, *Bifidobacterium*, and *Lactobacillus* in fecal samples. Data are presented as the mean ± SEM, *n* = 6. Different lowercase letters above the bars denote statistically significant differences (*p* < 0.05). The absence of a letter difference indicates no statistical significance *(p* > 0.05).

As shown in Figure 3A–D, compared to the NC group, the number of *E. coli* in the feces of the ETEC group was significantly increased (*p* < 0.05), while the numbers of *Lactobacillus* and *Bifidobacterium* were significantly decreased (*p* < 0.05). Compared to the ETEC group, both the HNa and HNa + CFM intervention groups showed a significant decrease in fecal *E. coli* count (*p* < 0.05) and a significant increase in the numbers of *Lactobacillus* and *Bifidobacterium* (*p* < 0.05). The combination of HNa and CFM significantly reduced the count of *E. coli* and increased the counts of beneficial bacteria (*Lactobacillus* and *Bifidobacterium*), a pattern that aligns with the classic features of a healthy gut microbiota.

### 3.4. HNa Combined with CFM Alleviates ETEC-Induced Impairment of Intestinal Proliferative Repair and Epithelial Cell Apoptosis in Mice

**Figure 4:**
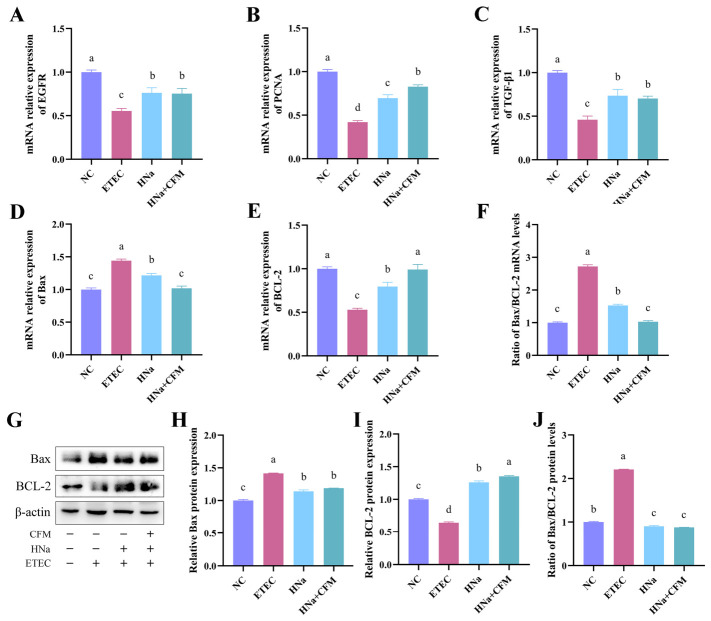
Effects of HNa Combined with CFM on Intestinal Proliferative Repair Capacity and Epithelial Cell Apoptosis in ETEC-Infected Mice. (**A**–**C**) Quantitative RT-PCR analysis of *EGFR*, *PCNA*, and *TGF-β1* mRNA levels in the jejunum; (**D**,**E**) Quantitative RT-PCR analysis of *Bax* and *BCL-2* mRNA levels in the jejunum; (**F**) Ratio of *Bax* to *BCL-2* mRNA levels; (**G**–**I**) Western blot analysis of Bax and BCL-2 protein levels in the jejunum, original Western blot images can be found in Figure S1; (**J**) Ratio of Bax to BCL-2 protein levels. Data are presented as the mean ± SEM, *n* = 6. Different lowercase letters above the bars denote statistically significant differences (*p* < 0.05). The absence of a letter difference indicates no statistical significance (*p* > 0.05).

To further validate the restorative effect of HNa combined with CFM on the intestinal barrier in ETEC-infected mice, the expression of factors related to epithelial cell proliferation was examined. Results in Figure 4A–C show that compared to the ETEC group, the mRNA expression levels of *EGFR*, *PCNA*, and *TGF-β1* in the jejunum were significantly increased in both the HNa and HNa + CFM groups (*p* < 0.05). Furthermore, compared to the NC group, the ETEC group exhibited a significant increase in the mRNA (Figure 4D) and protein (Figure 4H) expression levels of the pro-apoptotic protein Bax, as well as in the Bax/Bcl-2 ratio (Figure 4F,J) (*p* < 0.05). Conversely, the mRNA (Figure 4E) and protein (Figure 4I) expression levels of the anti-apoptotic protein Bcl-2 were significantly decreased (*p* < 0.05). Compared to the ETEC group, both HNa alone and HNa combined with CFM intervention significantly reduced the mRNA and protein expression of Bax and the Bax/Bcl-2 ratio (*p* < 0.05), while significantly increasing the mRNA and protein expression of Bcl-2 (*p* < 0.05). These results indicate that the intervention with HNa combined with CFM reversed the trend of intestinal epithelial cell apoptosis and worked to repair the intestinal barrier.

### 3.5. HNa Combined with CFM Inhibits the LPS-TLR4-NF-κB Pathway and Alleviates ETEC-Induced Inflammation in Mice

**Figure 5:**
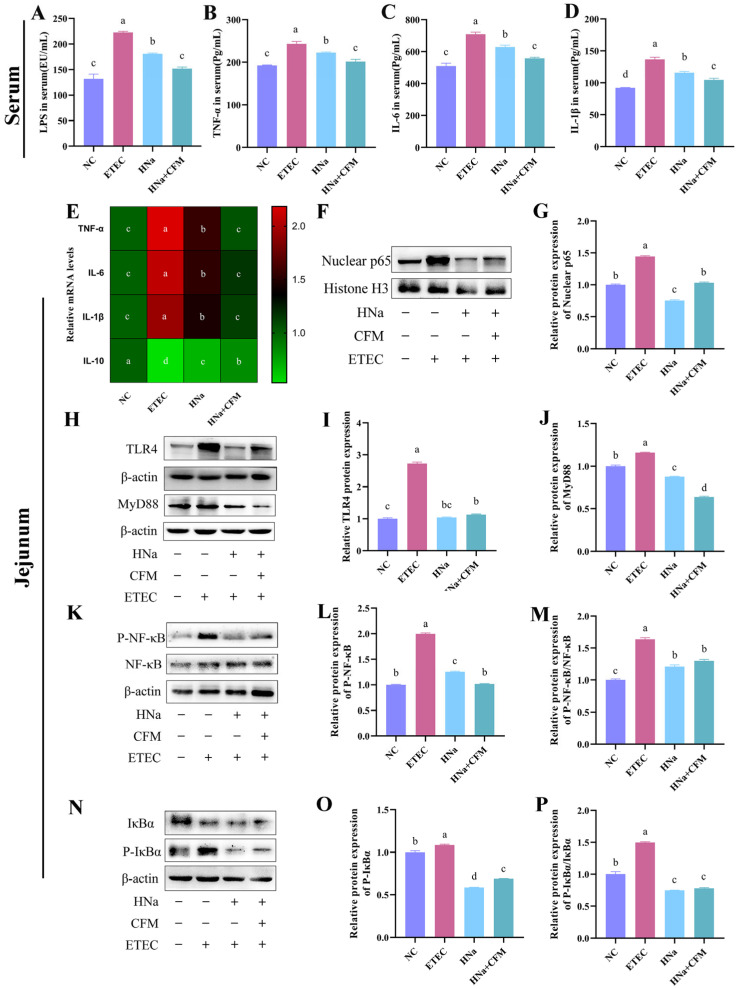
Effects of HNa Combined with CFM on Inflammatory Factors in ETEC-Infected Mice. (**A**–**D**) Levels of LPS, TNF-α, IL-6, and IL-1β in mouse serum. (**E**) Heatmap showing the relative mRNA expression levels of *TNF-α*, *IL-6*, *IL-1β*, and *IL-10* in jejunal tissue. (**F**–**J**) Western blot analysis and quantification of nuclear NF-κB p65, TLR4 and MyD88 protein expression. (**H**–**J**) Western blot analysis and quantification of TLR4 and MyD88 protein expression. (**K**–**M**) Western blot analysis and quantification of phosphorylated P-NF-κB p65 and the P-NF-κB/NF-κB ratio. (**N**–**P**) Western blot analysis and quantification of phosphorylated P-IκBα and the P-IκBα/IκBα ratio. Original Western blot images can be found in Figure S1. Data are presented as the mean ± SEM, *n* = 6. Different lowercase letters above the bars denote statistically significant differences (*p* < 0.05). The absence of a letter difference indicates no statistical significance (*p* > 0.05).

LPS, a major component of the cell wall of Gram-negative bacteria, is a classic activator of the TLR4-NF-κB pathway. As shown in Figure 5A, serum LPS levels were significantly increased in the ETEC group compared to the NC group (*p* < 0.05). Relative to the ETEC group, LPS levels were significantly reduced in both the HNa and HNa + CFM groups (*p* < 0.05). The combined intervention of HNa and CFM resulted in a more pronounced reduction in LPS levels compared to HNa treatment alone (*p* < 0.05). Concurrently, HNa intervention significantly suppressed the ETEC-induced excessive increase in serum levels of TNF-α, IL-6, and IL-1β (Figure 5B–D) (*p* < 0.05), and the suppressive effect was more significant with the combined HNa and CFM intervention (*p* < 0.05). Furthermore, in jejunal tissue (Figure 5E), compared to the NC group, the ETEC group showed significantly increased levels of *TNF-α*, *IL-6*, and *IL-1β* (*p* < 0.05) and a significantly decreased level of *IL-10* (*p* < 0.05). Compared to the ETEC group, both the HNa and HNa + CFM groups exhibited significantly lower levels of *TNF-α*, *IL-6*, and *IL-1β* (*p* < 0.05) and a significantly higher level of *IL-10* (*p* < 0.05). The improvement was more significant in the HNa + CFM group compared to the HNa group alone (*p* < 0.05). These results indicate that intervention with HNa combined with CFM can markedly reduce the levels of pro-inflammatory factors and increase the level of the anti-inflammatory factor in both serum and jejunal tissue. As shown in Figure 5F–P, ETEC challenge significantly increased nuclear NF-κB p65 accumulation, upregulated TLR4 and MyD88 protein expression, and enhanced the phosphorylation of NF-κB p65 and IκBα compared with the NC group, suggesting activation of the TLR4/MyD88/NF-κB signaling pathway in jejunal tissue. HNa alone and HNa combined with CFM significantly attenuated these ETEC-induced changes. Notably, the combined treatment reduced NF-κB p65 nuclear translocation, suppressed TLR4 and MyD88 expression, and inhibited NF-κB p65 and IκBα phosphorylation, and these effects were associated with inhibition of the TLR4/MyD88/NF-κB signaling pathway.

## 4. Discussion

ETEC is a major pathogen causing diarrhea in humans (particularly children and travelers) and young animals, and is responsible for substantial economic losses in the livestock industry [18]. Extensive research has demonstrated that ETEC infection directly induces intestinal damage and dysfunction, and can trigger systemic inflammatory and immune responses [19]. The long-term use of antibiotics has contributed to increasing resistance rates, thereby complicating treatment [20]. HNa possesses biological properties including anti-inflammatory, antimicrobial, and mucosal barrier-repair activities, along with advantages such as high ecological safety, favorable cost-effectiveness, and strong accessibility. Consequently, it has emerged as a potential therapeutic candidate for various diseases in both animals and humans [9]. However, monotherapy with HNa is limited by suboptimal efficacy and a relatively slow onset of action. CFM rapidly eliminates Gram-negative bacteria such as ETEC by inhibiting bacterial cell wall synthesis, leading to a prompt reduction in pathogen load [21]. Based on previous mouse-model PK/PD studies, a single oral dose of CFM of 12 mg/kg effectively cleared the extended-spectrum cephalosporin-susceptible FA1090 strain, whereas even 300 mg/kg CFM administered three times daily every 8 h cleared only 50% of infections caused by the resistant H041 strain [22]. In the present study, we employed a low-dose CFM regimen (2 mg/kg, orally, once daily for 3 consecutive days) in combination with HNa to evaluate the effect of the combined treatment on infection. In a mouse thigh infection model, oral cephalosporins tested at 2.5–320 mg/kg every 4 h required approximately 40–50% and 45–75% free-drug T > MIC to achieve stasis and 1-log kill against *E. coli*, respectively [23]; Nuding et al. reported in vitro that low-dose β-lactams alone have limited effect and require antimicrobial peptides [24]; Shan Q et al. demonstrated in neutropenic mice that cefquinome activity depends on maintaining %fT > MIC, with low doses ineffective. Therefore, 2 mg/kg CFM likely has minimal direct antibacterial effect, and the absence of a monotherapy control remains a limitation for quantifying its independent contribution [25]. In this study, compared to HNa monotherapy, the combination of HNa and CFM significantly reduced the abundance of fecal *E. coli*. It alleviates the inflammatory response in mice by inhibiting the LPS-TLR4-NF-κB pathway, reduces epithelial cell apoptosis, and restores intestinal proliferative repair function, ultimately leading to the rapid restoration of intestinal barrier structure. This strategy is particularly suitable for diseases such as ETEC infection, which require both acute infection control and long-term barrier repair. It provides a new perspective for addressing drug-resistant bacterial infections and comprehensive intestinal management in both clinical and livestock settings.

The intestinal barrier primarily consists of the chemical, mechanical, biological, and immune barriers [26]. The mucus layer is a component of the chemical barrier and is primarily composed of mucins secreted by intestinal epithelial cells [27]. Both tight junctions and adherens junctions are intercellular junctional structures between intestinal epithelial cells and serve as key components of the intestinal mechanical barrier [28]. In patients diagnosed with enteritis, structural damage and functional impairment are commonly observed in the intestinal mucus layer, tight junctions, and adherens junctions [29]. Studies have reported that ETEC infection can significantly downregulate the expression of MUC2 in the intestinal mucus layer and disrupt the distribution of tight junction proteins, leading to impaired intestinal barrier integrity and increased permeability [30]. Our results are consistent with these findings: ETEC infection induced structural damage to the ileal mucus layer in mice, suppressed the expression of genes related to both tight and adherens junctions, caused widening of the intercellular spaces between intestinal epithelial cells, and resulted in intestinal structural damage. Consistent with prior findings, our study confirms the beneficial effect of HNa on intestinal barrier repair in ETEC-infected mice [13]. Importantly, the combination of HNa and CFM demonstrated a superior therapeutic efficacy.

The intestinal biological barrier is fundamentally composed of the resident commensal microbiota, which includes beneficial bacteria such as *Bifidobacterium* and *Lactobacillus*, as well as dominant core phyla like *Bacteroidetes* and *Firmicutes* [31]. ETEC alters the patient’s intestinal microbiota structure either directly or indirectly through its specific fimbriae and secreted enterotoxins [32]. Broad-spectrum antibiotics are effective for treating refractory enteritis, yet they often carry the risk of disrupting the intestinal microecology [33]. This study adopted a therapeutic regimen combining HNa with CFM. In this regimen, the addition of low-dose CFM to HNa helps reduce ETEC load, while HNa increases beneficial bacteria such as *Bifidobacterium* and *Lactobacillus*, resulting in additional beneficial effects. This strategy not only helps overcome the limitations of single-antibiotic therapy but also provides a new perspective for effectively controlling infection while maintaining microbiota homeostasis in clinical practice. This approach is supported by prior research. For instance, the combined use of humic acid and lincomycin has been shown to improve the growth performance of broilers infected with *Clostridium* [15]. Furthermore, pectin oligosaccharides have been demonstrated to restore the microbiota balance and enrich specific beneficial bacteria in colitis mice, thereby enhancing antibiotic efficacy [34]. Collectively, these findings indicate that the targeted combination of microecological modulators with antibiotics holds broad application prospects for managing infectious diseases. A limitation of this study is that we did not perform 16S rRNA sequencing, and our qPCR analysis was limited to three bacterial groups, so the broader impact on gut microbiota composition remains to be characterized.

EGFR plays a crucial role in physiological processes such as cell growth, proliferation, and differentiation [35]. Furthermore, PCNA is closely associated with cellular DNA synthesis and serves as an important indicator of the proliferative state due to its key function in cell proliferation [36]. HNa has been shown to promote epithelial cell proliferation. For instance, a study by Ji et al. found that HNa facilitates rat skin wound healing by activating the TGF-β/Smads signaling pathway [37]. The results of the present study revealed that intervention with HNa combined with CFM significantly upregulated the mRNA expression of *EGFR*, *PCNA*, and *TGF-β1* in the mouse jejunum. This suggests that the combined intervention of HNa and CFM promotes the proliferation of intestinal epithelial cells. Intestinal epithelial cells are the primary target of *E. coli*, and inhibiting their apoptosis can effectively mitigate intestinal barrier damage [38]. Previous research has indicated that infection with pathogenic *E. coli* induces apoptosis in intestinal epithelial cells, leading to increased intestinal permeability and impaired barrier function [5]. In the present study, the expression of the pro-apoptotic protein Bax was significantly increased, while the expression of the anti-apoptotic protein Bcl-2 was significantly decreased in the jejunum of mice in the ETEC group, resulting in a markedly elevated Bax/Bcl-2 ratio. This demonstrates that ETEC infection triggers apoptosis in intestinal epithelial cells. The combined intervention of HNa and CFM reversed this condition and significantly reduced the Bax/Bcl-2 ratio. In summary, the combination of HNa and CFM alleviates ETEC-induced damage to intestinal epithelial cells by downregulating the expression of pro-apoptotic proteins, upregulating the expression of anti-apoptotic proteins, and promoting the expression of genes associated with cell proliferation.

LPS is a component of the outer membrane of Gram-negative bacterial cell walls. When the intestinal mucus layer is compromised, LPS can damage intestinal epithelial cells and translocate into the systemic circulation, thereby inducing the expression of inflammatory mediators such as TNF-α and IL-6, and triggering local and systemic inflammation [39]. Studies have shown that ETEC infection can lead to a significant increase in blood LPS levels [40]. This study found that serum levels of LPS, TNF-α, IL-6, and IL-1β were significantly elevated in ETEC-infected mice. However, following combined intervention with HNa and CFM, LPS levels markedly decreased and approached those of the negative control group, while TNF-α, IL-6, and IL-1β were also significantly reduced. Notably, serum levels of LPS and pro-inflammatory factors in the combined intervention group were significantly lower than those in the HNa-alone intervention group. This effect is likely attributable to the effective suppression of ETEC by CFM.

Research indicates that TNF-α is a critical regulator of the internalization of the tight junction protein Occludin, and a reduction in Occludin directly contributes to increased intestinal permeability [41]. In the context of enteritis, the overexpression of TNF-α and IL-1β not only exacerbates neutrophil infiltration but also directly impairs barrier structure [42]. Furthermore, cytokines such as IL-1β and IL-6 can downregulate the expression of tight junction proteins through an AMPK-dependent pathway [43]. Analysis revealed that in jejunal tissue, the combined use of HNa and CFM significantly reduced the mRNA levels of pro-inflammatory factors and significantly increased the level of the anti-inflammatory factor *IL-10*. Collectively, this evidence further substantiates that the combination of HNa and CFM maintains intestinal barrier integrity more effectively than HNa monotherapy. When the integrity of the intestinal barrier is compromised, pathogenic bacteria or their LPS can translocate into intestinal epithelial cells. This activates Toll-like receptor (TLR) signaling pathways upon binding, thereby exacerbating the inflammatory response [44]. Among these, TLR4, a key member of this receptor family, is potently activated by LPS. This activation initiates a transcriptional cascade centered on NF-κB, driving the inflammatory process [45]. This study found that ETEC infection markedly activated the TLR4/MyD88/NF-κB signaling pathway in the mouse jejunum, as evidenced by increased TLR4 and MyD88 protein expression, enhanced phosphorylation of NF-κB p65 and IκBα, and elevated nuclear accumulation of NF-κB p65. HNa alone and HNa combined with low-dose CFM significantly attenuated these ETEC-induced changes. Notably, the combined intervention further reduced intestinal *E. coli* burden and inflammatory activation compared with HNa alone. Intervention with the combination of HNa and CFM markedly suppressed this upregulation. In summary, the combined therapy reduced intestinal ETEC burden, effectively reducing LPS-triggered activation of the TLR4/NF-κB signaling pathway. Consequently, it alleviated ETEC-induced inflammatory damage at both local and systemic levels. These results suggest that HNa combined with low-dose CFM alleviates ETEC-induced jejunal inflammation, and this effect is associated with reduced bacterial burden and suppression of LPS-related activation of the TLR4/MyD88/NF-κB pathway, thereby contributing to the improvement of both local intestinal and systemic inflammatory responses.

## 5. Conclusions

This study indicates that, compared with HNa alone, the combined administration of HNa and low-dose CFM exerted a more pronounced protective effect on mice infected with ETEC. This combined strategy not only reduced intestinal pathogen burden but also promoted intestinal barrier repair through multiple mechanisms. The combination of HNa and CFM significantly ameliorated jejunal histopathological and ultrastructural damage, restored intestinal mechanical barrier integrity, modulated the balance of pathogenic and beneficial gut bacteria, promoted intestinal epithelial cell proliferation, and inhibited apoptosis. Its mechanism of action is closely associated with the inhibition of the LPS-TLR4-NF-κB signaling pathway and the alleviation of local and systemic inflammatory responses.

## Data Availability

All data generated or analyzed during this study are included in this article/Supplementary Materials. Further inquiries can be directed to the corresponding author.

## Supplementary Materials

The following supporting information can be downloaded at: https://www.mdpi.com/article/10.3390/biom16060814/s1, Table S1: Sequences of Primers for Genes; Table S2: Microbe-specific primer sequences, Figure S1: original Western blot images.

## Abbreviations

The following abbreviations are used in this manuscript:

HNa	sodium humate
CFM	cefixime
ETEC	enterotoxigenic *Escherichia coli*
DAO	diamine oxidase
D-lac	D-lactate
H&E	hematoxylin and eosin staining
MUC	Mucin
TEM	transmission electron microscope
EGFR	epidermal growth factor receptor
PCNA	proliferative cell nuclear antigen
TGF-β1	transforming growth factor-β1
Bax	BCL2-Associated X protein
BCL-2	B-cell Lymphoma 2
LPS	lipopolysaccharide
TNF-α	tumor necrosis factor-alpha
IL	interleukin
TLR4	Toll-like receptor 4
NF-κB	nuclear factor kappa-B
MyD88	Myeloid differentiation primary response 88
IκBα	Inhibitor of κB alpha
ZO-1	Zonula occludens-1
