# Supplementary material for: Sodium Humate Combined with Low-Dose Cefixime Alleviates Intestinal Injury in ETEC Infection via Inhibition of the TLR4/NF-κB Pathway
Source: Biomolecules. 2026 May 30;16(6):814. doi: 10.3390/biom16060814 (PMC13297103; doi:10.3390/biom16060814)
Supplement: Supplementary file 1 [file biomolecules-16-00814-s001.zip › biomolecules-4302125-supplementary.pdf]

Table S1 Sequences of Primers for Genes

Gene	Forward Primer (5'-3')	Reverse Primer (5'-3')
MUC1	GGCATTCGGGCTCCTTTCTT	TGGAGTGGTAGTCGATGCTAAG
MUC2	AGGGCTCGGAACTCCAGAAA	CCAGGGAATCGGTAGACATCG
MUC3	GCCGTGAATTGTATGAACGGA	CGCAGTTGACCACGTTGACTA
E-cadherin	CAGGTCTCCTCATGGCTTTGC	CTTCCGAAAAGAAGGCTGTCC
β-catenin	ATGGAGCCGGACAGAAAAGC	CTTGCCACTCAGGGAAGGA
ZO-1	AGCGAACAGAAGGAGCG	CTGCCAAACTATCTTGTGAAA
Occludin	GGTCTCTACGTGGATCAATATTTGTA	AACCCCAGGACAATGGCTA
Claudin-1	AGCACCGGGCAGATACAGT	GCCAATTACCATCAAGGCTCG
IL-1β	TTGACGGACCCCAAAAGAT	AGCTGGATGCTCTCATCAGG
IL-6	ACTTCCATCCAGTTGCCTTCTTG	AGCTGGATGCTCTCATCAGG
IL-10	GCTCTTACTGACTGGCATGAG	CGCAGCTCTAGGAGCATGTG
TNF-α	CATCTGAACTTCGGGGTGAT	CAGGCTTGTCACTCGAATT
PCNA	TTTGAGGCACGCCTGATCC	GGAGACGTGAGACGAGTCCAT
TGF-β1	CTCCCGTGGCTTCTAGTGC	GCCTTAGTTTGGACAGGATCTG
EGFR	GCCATCTGGGCCAAAGATACC	GTCTTCGCATGAATAGGCCAAT
Bax	TGAAGACAGGGGCCTTTTTG	AATTCGCCGGAGACACTCG
BCL-2	ATGCCTTTGTGGAACTATATGGC	GGTATGCACCCAGAGTGATGC
β-actin	GGCTGTATTCCCCTCCATCG	CCAGTTGGTAACAATGCCATGT

Table S2 Microbe-specific primer sequences

Items	Primer sequence (5'-3')	Product size (bp)
General bacteria	F：CGGCAACGAGCGCAACCC<br>R：CCATTGTAGCACGTGTGTAGCC	130
Escherichia coli	F：CATGCCGCGTGTATGAAGAA<br>R：CGGGTAACGTCAATGAGCAAA	96
Bifidobacterium	F：GATTCTGGCTCAGGATGAACGC<br>R：CTGATAGGACGCGACCCCAT	230
Lactobacillus	F：AGCAGTAGGGAATCTTCCA<br>R：CACCGCTACACATGGAG	341

Figure S1. Original Western blot images.

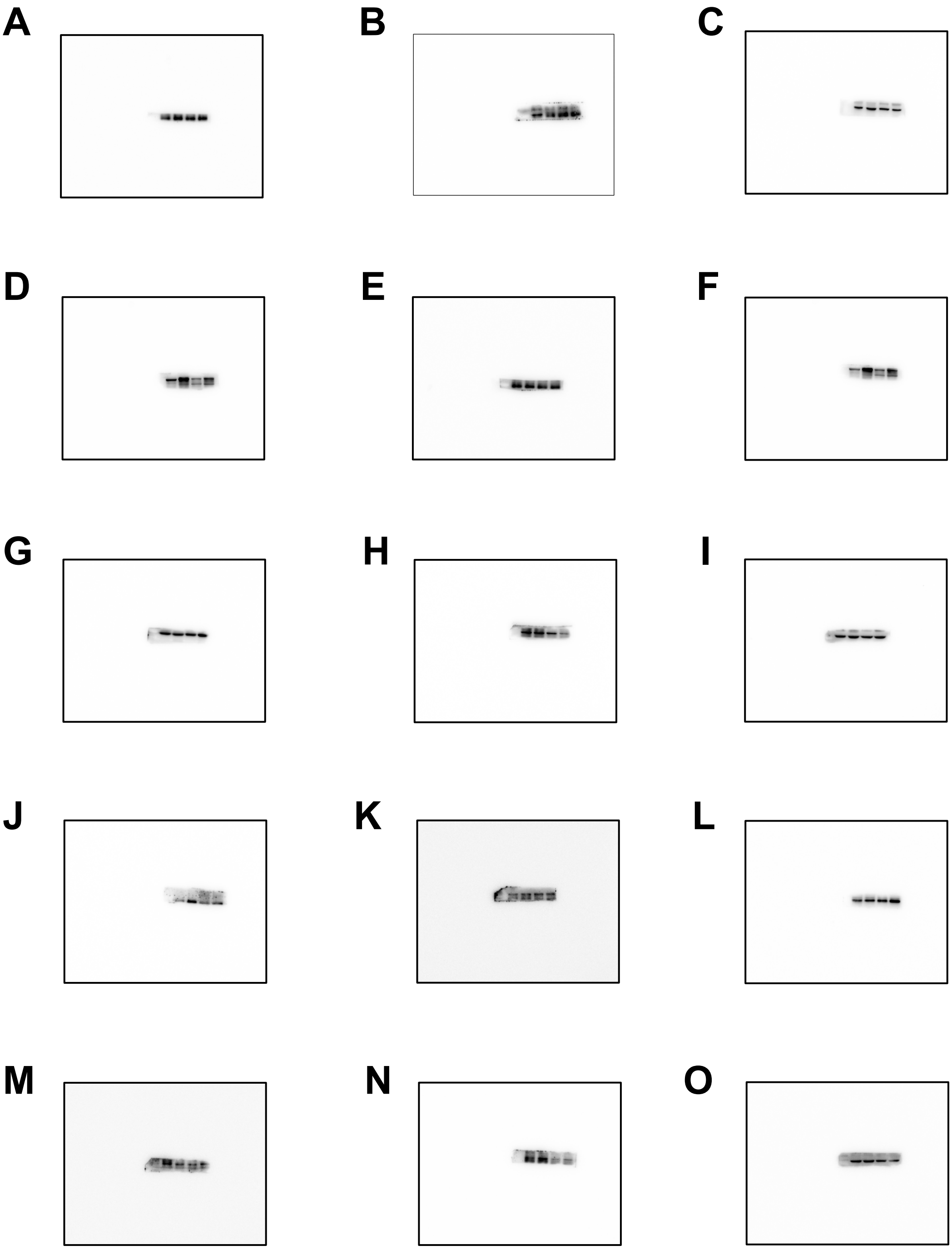

Figure S1. Original Western blot images. (A) Original blots for Bax. (B) Original blots for BCL-2. (C) Original blots for β-actin (Bax and BCL-2). (D) Original blots for Nuclear NF-κB p65. (E) Original blots for Histone H3. (F) Original blots for TLR4. (G) Original blots for β-actin (TLR4). (H) Original blots for MyD88. (I) Original blots for β-actin (MyD88). (J) Original blots for P-NF-κB p65. (K) Original blots for NF-κB p65. (L) Original blots for β-actin (P-NF-κB and NF-κB). (M) Original blots for IκBα. (N) Original blots for P-IκBα. (O) Original blots for β-actin (IκBα and P-IκBα).
